# Synthesis and spectroscopic studies of functionalized graphene quantum dots with diverse fluorescence characteristics

**DOI:** 10.1039/c8ra01148f

**Published:** 2018-03-22

**Authors:** Varun A. Chhabra, Rajnish Kaur, Naveen Kumar, Akash Deep, Changanamkandath Rajesh, Ki-Hyun Kim

**Affiliations:** Centre for Development of Advanced Computing(C-DAC) Phase VIII Mohali 160071 India; Sri Guru Granth Sahib World University (SGGSWU) Fatehgarh Sahib 140406 India rajeshc@sggswu.org; Department of Physics, Panjab University Sector 14 Chandigarh 160014 India; Central Scientific Instruments Organization (CSIR-CSIO) Sector 30 C Chandigarh 160030 India dr.akashdeep@gmail.com; Department of Civil and Environmental Engineering, Hanyang University 222 Wangsimni-Ro Seoul 04763 Korea kkim61@hanyang.ac.kr

## Abstract

In this research, we report a facile method for synthesizing a series of carboxyl functionalized graphene quantum dots (GQDs) using graphite flakes (300 meshes) as raw material. These highly luminescent GQDs emitted blue, light blue, green, yellow, and red light (400–700 nm intensity peaks) under ultraviolet irradiation conditions, while exhibiting quantum yields in the range of 50–70%. The products were comprehensively characterized using ultraviolet-visible, photoluminescence, infrared, Raman, and dynamic light scattering spectroscopies. The GQDs were found to remain highly stable against photobleaching when stored over a prolonged period of more than three months. The proposed method for the synthesis of high quality, multicolor GQDs can be utilized to extend the application of these nanoparticles to molecular biotechnology and bioengineering; for example, the immobilization of cancer markers on their surface. As such, carboxylic acid groups present on the surface of these GQDs help create complexes for *in vivo* sensing applications.

## Abstract 2

In this research, we report a facile method for synthesizing a series of carboxyl functionalized graphene quantum dots (GQDs) using graphite flakes (300 meshes) as raw material.

## Graphical abstract

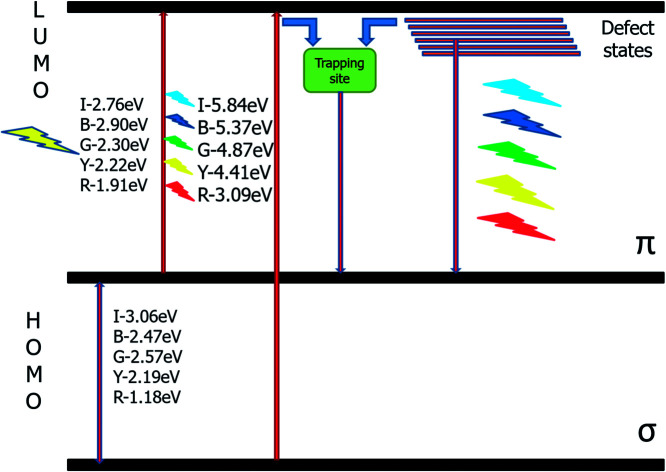

## 1. Introduction

Single-layer graphene is a single-atom-thick material with a unique structure comprised of a honeycomb lattice with atoms arranged in sp^2^ hybridization. Graphene is characterized by excellent electronic properties. Graphene structures have also been demonstrated to show photoluminescent properties by tuning of the band gap.^1–4^ Such modifications can be made possible by creating edge defects or *via* tuning of the shape and size of the material.^5^

In recent years, graphene quantum dots (GQDs) have rapidly emerged as one of the most prominent luminescent carbon nanomaterials.^6^ They exhibit some advantageous properties compared to other fluorescent nanoparticles (*e.g.*, improved biocompatibility, excellent photostability, low cytotoxicity, and confined emission of energy). As GQDs are capable of emitting two photons in a single excitation event, they can produce coherent light, which is important in the generation of lasers.^7,8^ Because of these advantageous properties, GQDs find applications in the fields of sensors,^9^ light (energy-from high to low frequency) conversion,^10^ photocatalysis,^11^ cell or bio imaging,^12,13^ and photovoltaics.^14^

GQDs can be synthesized *via* either “top-down” or “bottom-up” approaches. However, most of the time, the former strategy is limited by various factors including poor control of product size, low quantum yield, tedious synthesis processes, and the need for special equipment. In these methods, a relatively low quantum yield of produced GQDs could restrict their utility in photovoltaic, optoelectronics, and sensing applications.^15–18^ The latter methods (bottom-up) are feasible to exert better control of the product's properties (*e.g.*, lattice dimensions, size distribution, and morphology). These methods basically involve the carbonization of organic precursors through thermal treatment.

The potential of GQDs for multicolor emissions has a number of technological significances and scientific interests.^10^ Once suitably developed, the extraordinarily attractive GQDs can be bound with other materials or components to further extend their application to such areas as optoelectronics, photovoltaics, and sensors.^19–21^ However, the synthesis of size controlled GQDs is a challenging task to meet conditions for the creation of surface emitting states. The available literature on the synthesis of multicolor GQDs mainly describes the doping of extraneous elements within the graphene hexagonal matrix.^22–24^ Generally, these methods involve treatment of GO with a mixture of NH_4_OH and H_2_O_2_ for long time intervals (such as 40, 120, and 270 min) to yield GQDs with different colors.

Numerous methods have been developed and employed to convert graphene-based materials into fluorescent GQDs. Further, the bandgap of GQDs can be tuned by changing the particle size and surface chemistry. Top-down approaches include hydrothermal (or solvothermal) cutting, electrochemical scissoring, nanolithography, microwave-assisted breaking, cage opening treatments of fullerene, and chemical exfoliation.^25–28^ GQDs prepared from these processes tend to exhibit poor solubility as they have heterogeneous structures and typically do not possess functional groups. On the other hand, bottom-up approaches are comparatively complex due to solution chemistry or carbonization and involve the use of organic solvents, which leads to a large quantity of waste. Bottom-up approaches include the assembly of GQDs from molecular precursors through hydrothermal methods, precursor pyrolysis, and metal catalyzed decomposition of materials like graphite, graphene sheets, and C60.^15,19,29,30^

As available information reveals, most of the reported methods for the synthesis of multicolor GQDs involve the application of sophisticated equipment, high-temperature synthesis conditions, or high-energy laser ablation. In this work, we propose a new method for the synthesis of graphene quantum dots that is highly advantageous in terms of simplicity, reproducibility, and high yield. In the present research, we demonstrate a much easier and highly effective method to synthesize multicolor GQDs with high quantum yields.

Table 1 lists the key points and novelty of the present method.

**Table 1:** Features of different processes reported for synthesis of GQDs

Order	Samples	Reference	Change	Reason
1	B-GQDs	58	High fluorescence toward a blue shift	High reaction temperature and dialyzed solution
2	I-GQDs	59	High fluorescence	Subsequent dialysis
3	G-GQDs	58	High yield	Small amount of oxidizing agent and lower reaction temperature
4	Y-GQDs	60	Improved upconversion properties	
5	R-GQDs	61	Low yield and feeble fluorescence	Facile but efficient synthesis with two functionalized groups in GQD solution

## 2. Experimental

### 2.1 Materials and equipment

The chemicals used in the course of the study included graphite flakes (300 mesh), sulfuric acid (H_2_SO_4_), potassium permanganate (K_2_MnO_4_), hydrogen peroxide (H_2_O_2_), hydrochloric acid (HCl), sodium hydroxide (NaOH), citric acid (CA), sodium nitrate (NaNO_3_), and urea. All of these chemicals with a high purity were purchased from Sigma (India)/Merck (India). Characterization of the synthesized materials was performed with various instruments: photoluminescence (PL) spectroscope (Varian Cary), Ultraviolet-Visible (UV-Vis) spectroscope (Varian Cary 5000), Fourier transform infrared (FTIR) spectroscope (Nicolet iS10), Raman spectroscope (Invia, Renishaw), and dynamic light scattering (DLS) spectroscope (Nano ZS90, Malvern).

### 2.2 Procedure

**Fig. 1:**
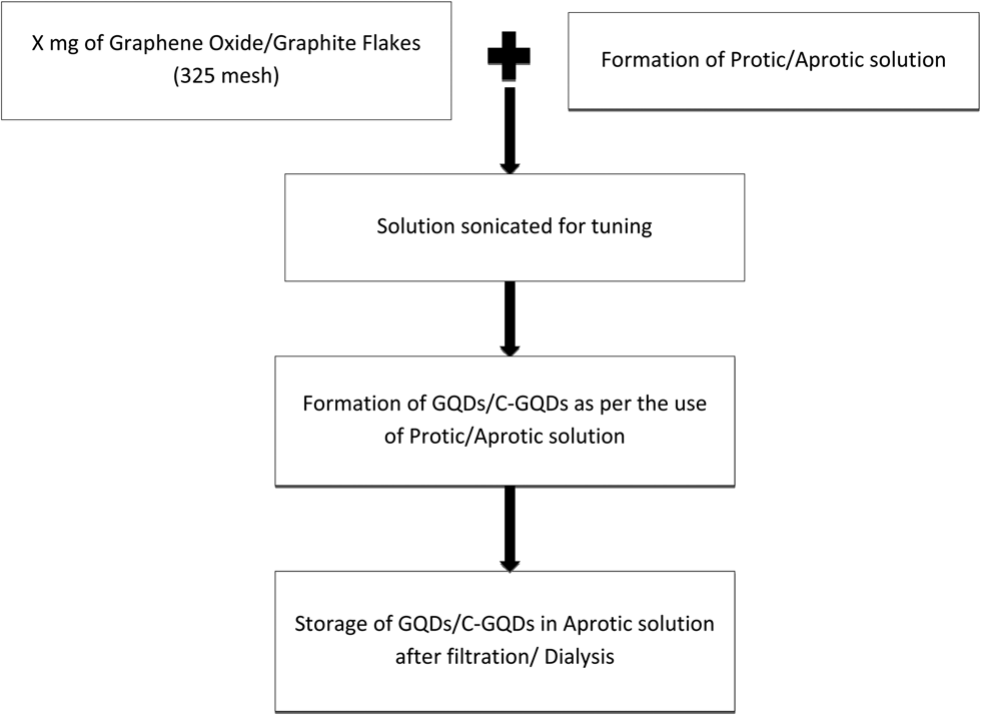
Flow chart for the generic methodology used for the formation of GQDs.

**Fig. 2:**
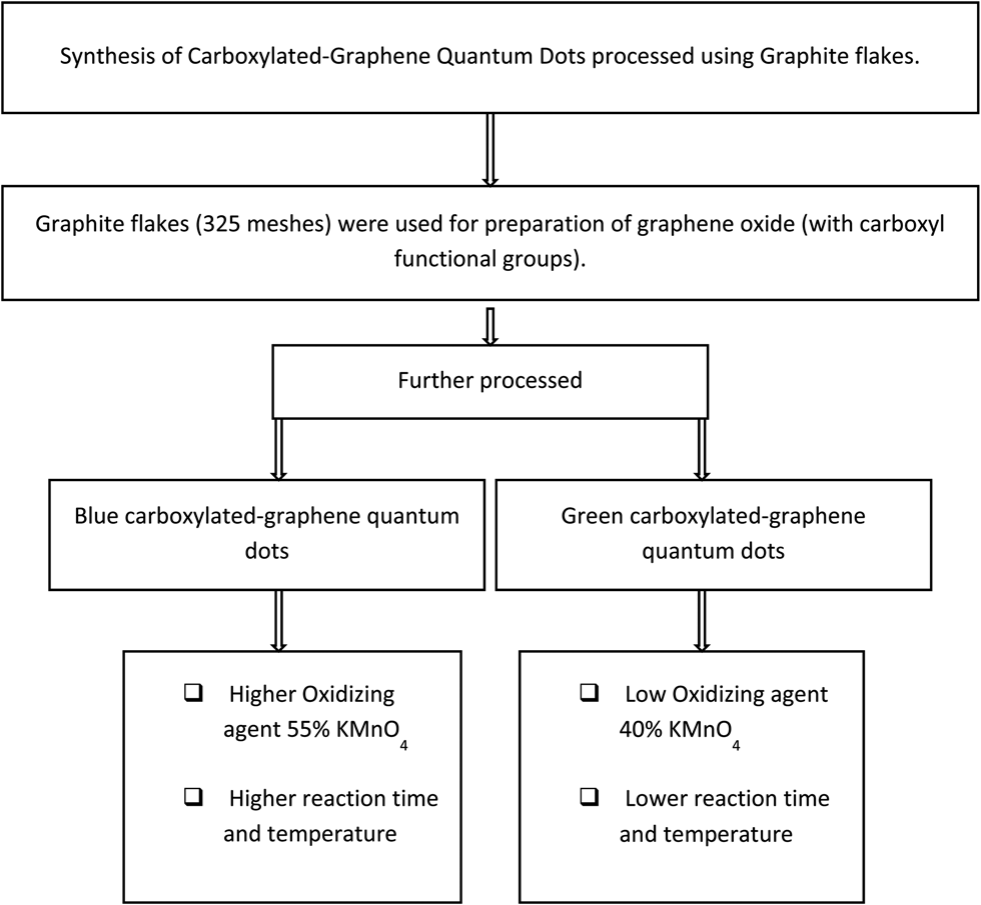
Flow chart summarizing the method used for production of B-GQDs and G-GQDs in this research.

Procedural details of the experiments conducted are explained further in this section; here Fig. 1 presents the generic methodology for the formation of GQDs, and the Fig. 2 flow chart shows the method for production of B-GQDs and G-GQDs.

#### 2.2.1 Preparation of graphene oxide

A modified Hummers' method was used for the preparation of graphene oxide. Briefly, H_2_SO_4_ (55 mL, 98%) and graphite flakes (1.0 g) were mixed in an ice bath, followed by slow addition (30 minutes) of 5.5 g of KMnO_4_ with continuous stirring. This solution was heated for 2 h at 30 °C, after which 50 mL of deionized water was added slowly. The reaction mixture was again heated at 90 °C for 30 minutes. Next, 80 mL of deionized water was added followed by the addition of 12 mL of 30% H_2_O_2_. As the temperature of the reaction mixture decreased to 60 °C, an orange colored solution was produced. Subsequently, an HCl solution (5%, 200 mL) was added. The supernatant, containing the desired graphene oxide, was separated, and the pH was adjusted to 4–6 by the addition of water. The solution was then subjected to dialysis in a dialysis bag for 2 days. Lyophilization of the dialyzed solution (at 21 Pa and −48 °C) resulted in the formation of graphene oxide in the form of a characteristic low density, yellow-grey colored, powdery material.

#### 2.2.2 Preparation of oxygen-rich graphene oxide (OGO)

Oxygen-rich graphene oxide (OGO) was also synthesized by employing the modified Hummers' method. For this, 2.5 g of graphite powder, 1.2 g of NaNO_3_, and 70 mL of concentrated H_2_SO_4_ were added to a 500 mL round bottom flask placed in an ice cooled sonicator bath. The mixture was sonicated for 20 minutes and then left undisturbed for another 2 hours (*T* = 4 °C). The contents were then transferred to another reaction vessel and heated to 85 °C for 3 hours with constant stirring. This mixture was subsequently diluted with 100 mL of deionized water and left to cool overnight. The next day, a small volume of 3% H_2_O_2_ was added to complete the solubilization of the reactants, which resulted in a change in color from a brown suspension to yellow. Next, low-speed centrifugation (5000 rpm) to remove any leftover impurities was followed by dispersion of the supernatant in 100 mL of 0.1 M HCl solution (2 hours). The pH of the solution was then adjusted to 7 with the addition of water. The above procedure yielded OGO.

#### 2.2.3 Preparation of blue emitting GQDs (B-GQDs)

For the synthesis of B-GQDs, 0.05 g of graphene oxide (as synthesized above, Section 2.2.1) was dispersed in 10 mL of concentrated H_2_SO_4_, and the mixture was left for 6 hours. Thereafter, the solution was mixed with 55 wt% of KMnO_4_ powder. The contents were stirred for 1 hour at room temperature (25 ± 2 °C, RT) and for another 1 hour at 80 °C. A small volume of 30% H_2_O_2_ was then added to ensure complete consumption of the KMnO_4_ powder in the mixture. Next, the reaction solution was poured over ice. The obtained solution was purified by filtration through a polytetrafluoroethylene (PTFE, 0.22 μm) membrane. The obtained blue colored GQD solution was further purified by dialysis.

#### 2.2.4 Preparation of indigo emitting GQDs (I-GQDs)

The synthesis of I-GQDs was carried out with citric acid (CA) as the main precursor. Briefly, 5 g of CA was placed in a 50 mL round bottom flask. A silicone oil bath was used to attain different temperatures (*i.e.* 170, 200, 220, and 250 °C). Then, 20 mL of NaOH solution (0.5 M) were added, and the mixture was stirred for 20 minutes. After dispersion of the solution, it was transferred to a 100 mL beaker followed by adjustment of the pH to 7.2. The obtained solution was subjected to dialysis with the aid of a 3.5 kDa tube membrane. After dialysis, the tube was immersed in 20 mL of deionized water for 24 h. The purified GQD solution (retained in the tube membrane) was again purified after transferring the contents into a 1 kDa dialysis tube membrane. The desired product was collected after dialysis for 24 h.

#### 2.2.5 Preparation of green emitting GQDs (G-GQDs)

Briefly, graphene oxide (1 g) was suspended in concentrated H_2_SO_4_ for about 2 h, followed by the addition of 40 wt% of KMnO_4_ powder. The acidic and oxidizing conditions provided shear-influenced exfoliation of the graphene oxide. This reaction solution was left under stirring conditions for 2 hours at RT. Next, the solution was heated for about 1 h at 45 °C. During this heating step, 40 mL of deionized water was added, resulting in effervescence. After 30 minutes, the temperature of the reaction mixture rose to about 80 °C. A small volume of 30% H_2_O_2_ was then added to ensure complete consumption of KMnO_4_ powder. The reaction was then quenched by pouring the reaction mixture over ice. The obtained solution (around 70 mL) was subjected to ultra-sonication for 5–6 min. As the solution cooled to RT, the pH was adjusted to around 8 with 10% NaOH solution, yielding flocculent black deposits. The pH was readjusted to 4 with the addition of 1 M HCl to yield a deep yellow colored solution. This solution was separated from the large particles of graphene by filtration through a microporous membrane (PTFE, 0.22 μm). Dialysis of the supernatant with the aid of a 3 kDa membrane allowed the attainment of G-GQDs.

#### 2.2.6 Preparation of yellow GQDs (Y-GQDs)

The synthesis of Y-GQDs began with OGO as the starting material (see Section 2.2. for the synthesis of OGO). In the process, 50 mL of OGO solution was transferred into an autoclavable mixer (100 mL), and the suspension was dispersed with high-speed shearing for 45 min. Subsequently, 10 mL of NaOH (1 mol L^−1^) was added into the autoclave and ultrasonically dispersed for 20 min. Next, the contents were transferred into a vessel for heating in an electronic oven at 200 °C. The solution was left to cool to room temperature, and filtration of the solution was carried out using a 0.22 μm microporous membrane. A yellow filtered solution was obtained using a dialysis bag, and the yellow solution was further dialyzed for 24 h, as it had a fluorescent substance, (retained molecular weight: 500 Da), and GQDs with strong fluorescence remaining in the bag were obtained in a yield of *ca.* 27%. Freeze drying was used to remove water from the GQD solution. Finally, a pure honeycomb of graphene quantum dots was obtained.

#### 2.2.7 Preparation of red GQDs (R-GQDs)

Graphene oxide was also used for the synthesis of R-GQDs. The sample of prepared GO (5 mL) was mixed with a mixture of 5 mL of 40% H_2_O_2_ and aniline (0.26 g), and the reaction was heated to 120 °C for 10 min. After allowing the solution to cool to room temperature, the contents were filtered using a 0.22 μm PTFE membrane. The supernatant was purified with 3 kDa dialysis membrane for 24 h, which resulted in the formation of R-GQDs with a strong fluorescent nature.

As elaborated in Section 2.2.3–2.2.7, the products of GQDs were purified each time with dialysis procedure. Note that such purification approach for GQDs (from their starting precursors) was reported to be employed in many earlier studies as well.^31–33^

## 3. Results & discussion

### 3.1 Spectroscopic characterization of GQDs samples

The formation of GQDs was a result of hydrothermal exfoliation of the raw materials (GO or CA). The application of potassium permanganate in acidic solution is associated with the oxidation of alkenes, which is the basis of the mechanism that results in the formation of G-GQDs and B-GQDs^34,35^ In this process, a manganate ester is first formed, which acts as the rate determining step. The formation of GQDs from this kind of mechanism is thoroughly explained in earlier literature.^36–41^ Thus, different sized GQDs can be obtained by varying the extent of the oxidation reaction.

Note that the carboxyl groups in the formed GQDs function as trapping sites or defect states, which can alter the LUMO energy states that influence the emission wavelengths even when the sample is excited by an unchanged UV energy wavelength. Thus, it is possible to obtain a range of visible spectra by varying the size of the synthesized GQDs. Similar to other carbon luminescent nanoparticles, the GQDs also show excitation-dependent photoluminescence behavior; a red shift was observed in the emissive wavelength when a lower wavelength energy was used for the excitation of samples.^42–44^ The GQDs prepared in this work also showed excitation-dependent photoluminescence behavior. The PL peak wavelengths were shifted at different excitation wavelengths due to the different scales of the quantum confinement effects with different nanoparticle sizes. A variation in the excitation energy led to changes in the intrinsic and extrinsic states of GQDs at different excitation energies. As a result, their PL spectra were found to be shifted.^45,46^

### 3.2 FTIR analysis of GQD samples

FTIR studies were carried out to study the presence of functional groups on the synthesized GQDs. The presence of –C=O groups was evident on both the starting (*i.e.*, graphene oxide) and final (GQDs) samples. Signals for the stretching vibration of –C–O–C (below 1250 cm^−1^) and absorption of C–H stretching vibrations (at 2950 cm^−1^) were also observed in G-GQDs. The G-GQD sample also displayed FTIR bands at around 1550 cm^−1^ (skeletal vibration of aromatic rings) and 3050 cm^−1^ (stretching vibration of C–H in aromatic rings).^13,25^ However, the B-GQD sample did not show any absorption of –C–O–C.

The I-GQD sample showed a band around 3320 cm^−1^ assigned to the hydroxyl (O–H) group stretching vibration, while the signal around 2900 cm^−1^ was attributed to symmetric and asymmetric stretching of C–H. The bending vibrations of the C=C group were related to an observed band at 1819 cm^−1^. The C–O (alkoxy), C–O (carboxy), and C=O (carboxyl) groups can be assigned to bands at 1389, 1660, and 2008 cm^−1^, respectively.

The FTIR spectra of Y-GQDs showed the presence of C=C bond vibration at 1661 cm^−1^, while a strong band at 3462.69 cm^−1^ can be assigned to hydroxyl (O–H) groups. The C–O stretching vibration was assigned to the band at 1235 cm^−1^. The presence of C–H and C–OH stretching vibrations was related to a signal at 1523.46 cm^−1^. As quantum dots formed after cleavage of sp^2^ domains of graphene oxide, the Y-GQD sample also showed the presence of –COOH and –OH groups.

**Fig. 3:**
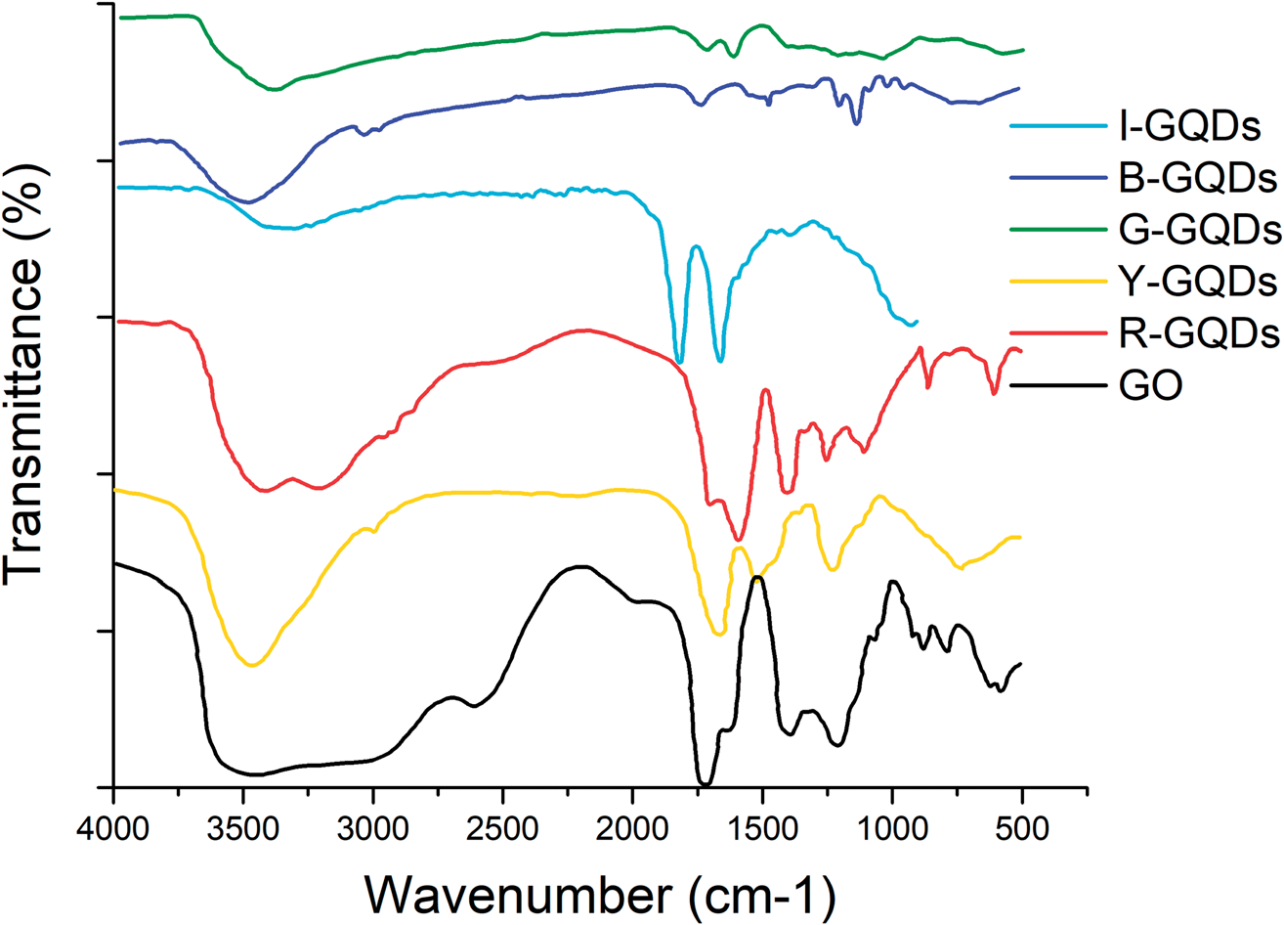
FTIR spectra of graphene quantum dot samples.

The FTIR spectra of R-GQDs showed the presence of C=C, C=O, and O–H bonds *via* the observation of bands around 1690, 1589, and 3416 cm^−1^, respectively. The stretching vibrations of oxygen-related groups in GQDs were much more intense than in GO.^47^ The appearance of a band around 1403 cm^−1^ was due to C–N absorption. The presence of a band around 3202 cm^−1^ was associated with the N–H stretching vibration of amine groups, demonstrating successful incorporation of nitrogen atoms (due to aniline) into the GQDs. The aqueous stability of different samples of synthesized GQDs was favored by the presence of carboxyl (–COOH) and hydroxyl (–OH) groups (Fig. 3).

### 3.3 Raman spectroscopic analysis

Raman spectroscopy is a vital technique to assess the quality of synthesized GQDs. The D and G band features reveal important information about the successful formation of the desired quantum dots. These bands are associated with disorder and defects in the hexagonal lattice (D band) and sp^2^ carbon atom vibrations (G band). The ratio of the intensity of these bands (*I*_D_/*I*_G_ ratio) is used to express the extent of sp^2^/sp^3^ hybridization of carbon atoms.^14,48^

**Fig. 4:**
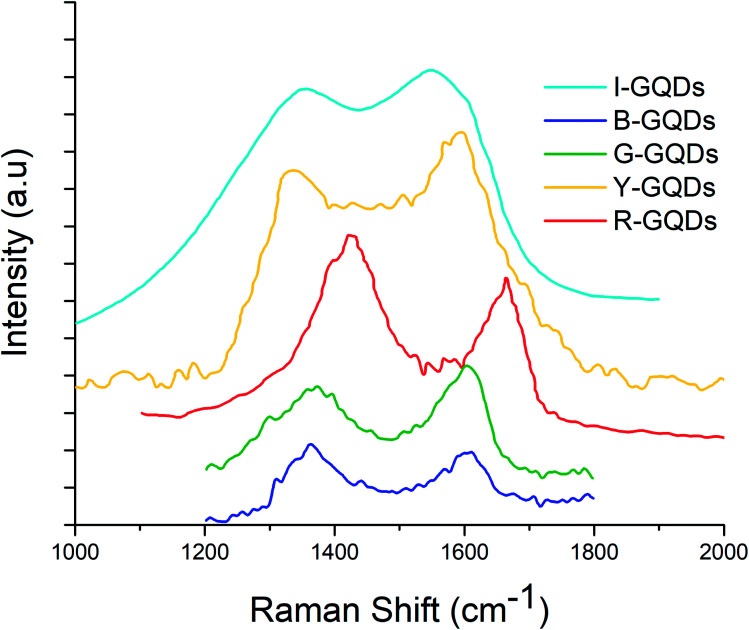
Raman spectra of graphene quantum dot samples.

Raman spectra of the synthesized G-GQDs and B-GQDs are shown in Fig. 4. The G-GQDs display the presence of ‘D’ (1367 cm^−1^) and ‘G’ (1611 cm^−1^) bands with a higher *I*_D_/*I*_G_ ratio (0.99) than B-GQDs (0.97). The peak positions of the D and G bands in B-GQDs were observed at 1372 and 1598 cm^−1^, respectively. The I-GQD sample also showed the presence of D (1352 cm^−1^) and G (1549 cm^−1^) bands, with an *I*_D_/*I*_G_ ratio of 0.86. The Y-GQDs were characterized by D and G bands at 1334 and 1597 cm^−1^, respectively, and an *I*_D_/*I*_G_ ratio of 0.89. Similarly, the R-GQD sample yielded characteristic D and G bands around 1442 and 1665.95 cm^−1^, respectively, with an *I*_D_/*I*_G_ value of 0.81. The Raman spectra of all synthesized GQDs displayed two prominent peaks about 1340 cm^−1^ and 1581 cm^−1^, which correspond to the D and G bands and confirm the successful synthesis of GQDs.^49^

### 3.4 UV-Vis absorption analysis

**Fig. 5:**
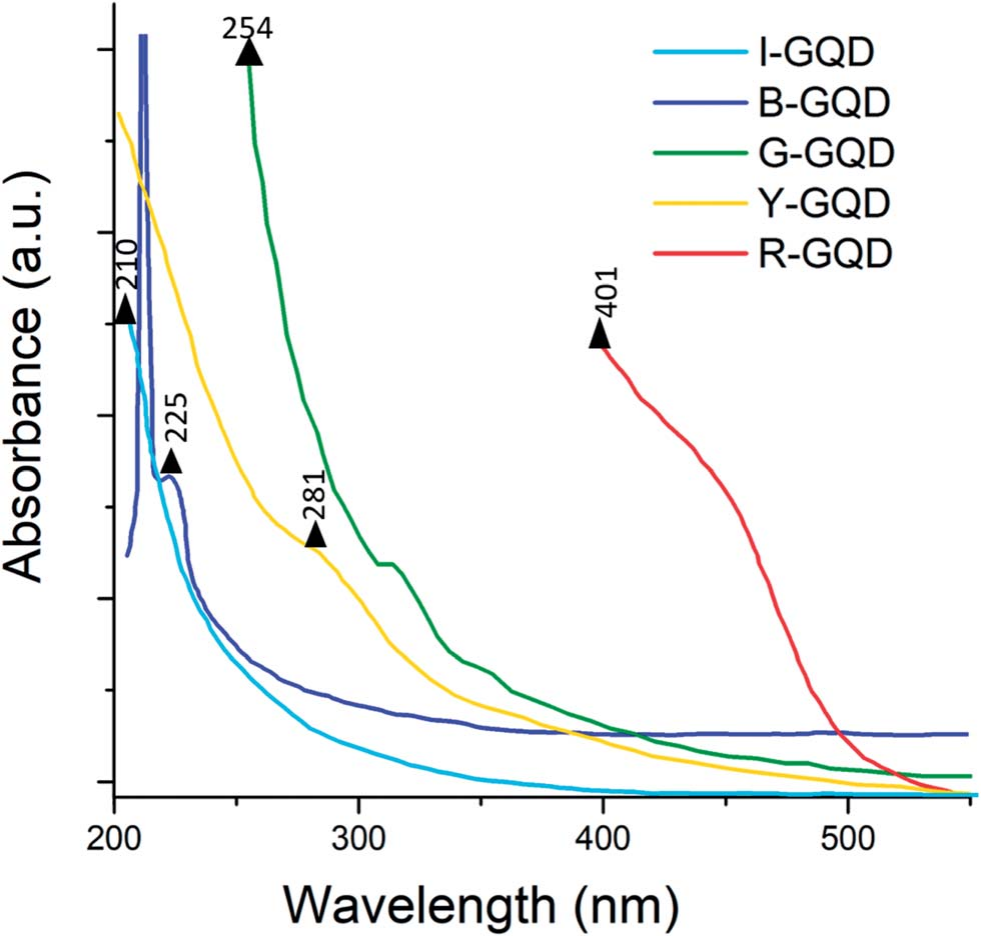
UV-Vis spectra of graphene quantum dot samples.

The light absorption profiles of the synthesized GQD samples are presented in Fig. 5. The G-GQDs exhibited peaks at 254 and 313 nm assigned to the π–π* transition of the aromatic sp^2^ domain. Absorption peaks for B-GQDs were observed at 212 and 225 nm. Note that the peak at 225 nm should represent the π–π* transition in this sample. The I-GQDs had an absorption peak at 210 nm. The Y-GQDs were characterized by a peak at 202 nm (assigned to π–π* transitions) and another peak at 281 nm (assigned to n–π* transitions).^50,51^ For the R-GQDs, the major absorption peaks were observed at 401 and 456 nm.

In GQDs, various electronic transition states are caused by the anti-bonding and bonding molecular orbitals of C=C sp^2^ carbon domains and functional groups with amino edges. These functionalizations are the reason for n–p and p*–p transitions. The regimes of lower wavelength transitions are dominated by p*–p primary transitions, which are suppressed at higher excitation wavelengths such that n–p transitions become dominant.^52^

### 3.5 Photoluminescence analysis

**Fig. 6:**
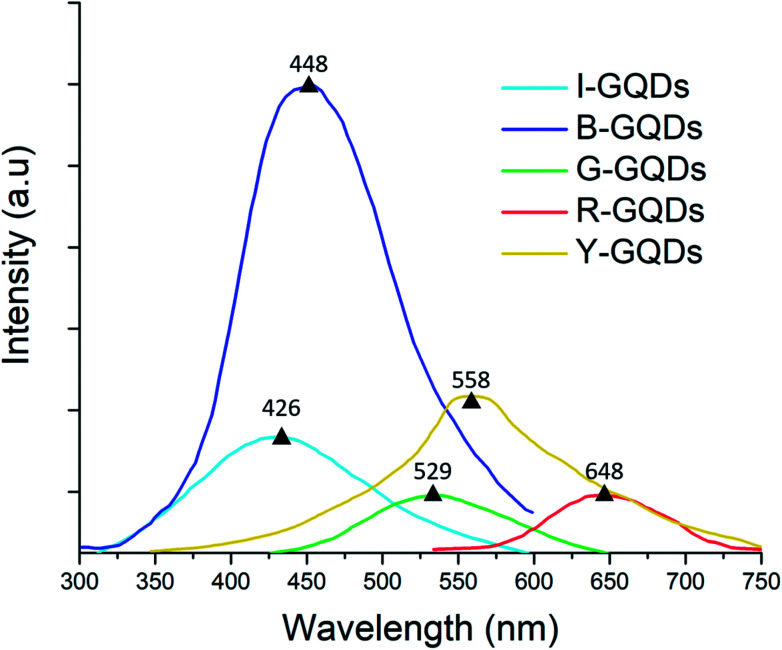
Photoluminescence spectra of graphene quantum dot samples.

Fluorescent carbon materials have a common property of showing excitation-dependent photoluminescence (PL) behavior. In the present studies, the G-GQD samples, when excited by energy of 254 nm wavelength, resulted in a PL peak at 539 nm (a strong green emission) (Fig. 6).

Likewise, in B-GQDs, the intrinsic state emission from defect states resulted in PL with an emission peak at 448 nm (samples excited by 212 nm energy). The I-GQDs, when excited at 231 nm, had an emission peak at 426 nm. The Y-GQDs (excitation energy = 281 nm) and R-GQDs (excitation energy = 401 nm) displayed emission peaks at 558 and 648 nm, respectively.^53–55^

The lower frequency wavelength was the result of an active process of two or multi-photon transitions with defined energy levels giving rise to the photoluminescence property.

### 3.6 Dynamic light scattering (DLS) analysis

**Fig. 7:**
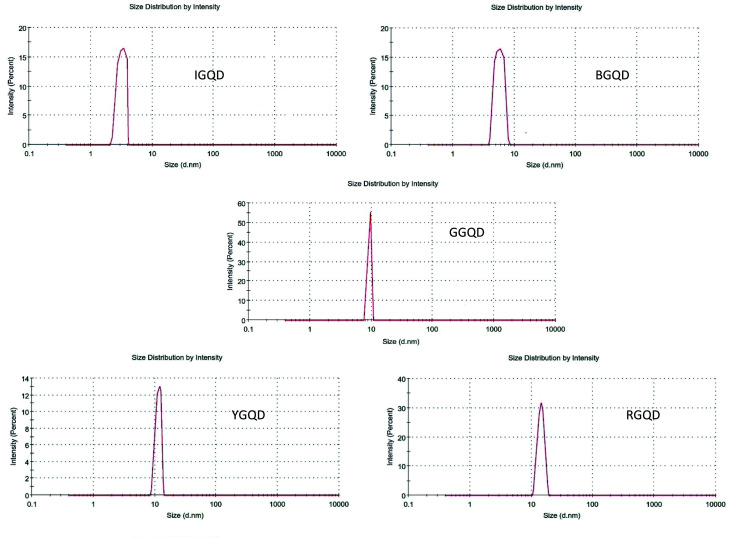
DLS spectra of fluorescent graphene quantum dots.

Results of the DLS analysis are shown in Fig. 7. The average particle sizes of the synthesized I-GQDs, B-GQDs, G-GQDs, Y-GQDs, and R-GQDs were observed to be around 3.245 nm, 6.952 nm, 10.02 nm, 13.72 nm, and 15.96 nm, respectively. These size determinations suggest that the emission properties of the GQDs were the result of (zigzag effect)/quantum size effect or recombination of holes and electrons in the quantum sized nanoparticles. In some instances, there also could be the possibility of two- or multi-photon emission, giving rise to a broad range of emissive wavelengths.^56,57^

In summary, the results of the present research are summarized in Table 2. The proposed synthetic methods for multicolored GQDs provide a relatively convenient and reproducible approach to achieve the formation of nanoparticles with the desired properties. The different colors of GQDs can be used for various purposes, such as to develop sensitive detection based on the recording of optical signals. The tunable PL response in GQDs should be very useful in biosensing applications, targeted drug delivery, cancer therapy, optoelectronics, and many other applications.

**Table 2:** Summary of the critical parameters for the synthesized GQDs based on spectroscopic techniques

Order	Samples	Raman peaks/bands (cm^−1^)	UV-Vis absorption peaks (nm)	PL emission peaks (nm)	DLS (average size) (nm)
1	I-GQDs	1361.98 (D)	210 nm	426.77	3.245
		1611.65 (G)			
2	B-GQDs	1352.12 (D)	212.45	448.72	6.952
		1549.44 (G)	225.12		
3	G-GQDs	1373.76 (D)	254.53	539.30	10.02
		1603.81 (G)	313.52		
4	Y-GQDs	1334.83 (D)	202.11	558.51	13.72
		1597.34 (G)	281.35		
5	R-GQDs	1424.92 (D)	401.40	648.63	15.96
		1665.95 (G)	456.91		

## 4. Conclusion

This aim of this study was to increase the fluorescence quantum yield by improving the conditions for preparing graphene quantum dots (GQDs) through the solvothermal route. The following experimental conditions for preparation of GQDs through the solvothermal route were improved: graphene oxide (GO)/*N-N* dimethyl formamide (DMF) ratio, filling percentage, and reaction time.

Currently, various techniques have been developed to produce GQDs, but they are complex and result in materials with lower fluorescence quantum yields and intensities compared to other quantum dots.

In our study, the fluorescence quantum yield of synthesized QDs reached up to 50%. The synthesis methods selected for the preparation of GQDs were modified for a simple, easy to control, and safe synthetic process. Overall, it was possible to increase the fluorescence quantum yield by improving the conditions for GQD preparation (Table 3). GQDs with a high quantum yield and strong photoluminescence show good biocompatibility and thus have good potential for cell imaging, biolabeling, and other biomedical applications.

**Table 3:** Summary of the quantum yields calculated for the synthesized GQDs

Order	Sample	Quantum yield (%)		References
		Present work	Earlier reported	
1	B-GQD	44.23	9.9	62
2			5	63
3			45	64
4	I-GQD	28	1.6	65
5			22.96	62
6	G-GQD	26	44.5	50
7			8	66
8	Y-GQD	34.62	9	50
9			23.6	60
10	R-GQD	21.52	1	61

## Conflicts of interest

There are no conflicts to declare.

## Supplementary Material
